# Monitoring Real-World Function With an Oura Ring Following Bilateral Subthalamic Nucleus Deep Brain Stimulation for Parkinson’s Disease

**DOI:** 10.7759/cureus.115385

**Published:** 2026-08-28

**Authors:** Andy Choi, Cristina Gonzalez-Robles, San San Xu, Marie T Krueger, Aswin Chari

**Affiliations:** 1 Medical School, University College London, London, GBR; 2 Department of Clinical and Movement Neurosciences, University College London (UCL) Queen Square Institute of Neurology, London, GBR; 3 Department of Neurosurgery, National Hospital for Neurology and Neurosurgery, London, GBR

**Keywords:** dbs for parkinson’s, deep-brain stimulators (dbs), digital device technology, digital technologies, parkinson`s disease, smart health wearable, wearable device

## Abstract

Subthalamic nucleus deep brain stimulation (STN DBS) is a well-established therapy for Parkinson’s disease (PD), but conventional follow-up may miss postoperative fluctuations. Consumer-grade wearable devices present a scalable, low-burden means of longitudinal monitoring outside the clinic. We report a single-patient case of a 68-year-old man with PD who continuously wore an Oura Ring before and after STN DBS. Proprietary composite “Activity”, “Readiness”, and “Sleep” scores were analysed descriptively across predefined 60-day pre- and post-DBS periods, excluding the immediate perioperative period. Longer-term trends were assessed over 12 months alongside clinical outcomes. Early post-DBS, "Sleep Score” improved from 45.0 to 53.5 and “Readiness Score” from 67.5 to 71.0, while “Activity Score” and daily step count remained stable despite improvement in the Movement Disorder Society-Unified Parkinson’s Disease Rating Scale Part III score. This divergence suggested that clinic-based motor improvement did not translate into increased free-living activity. “Sleep” and “Readiness” scores remained higher during later follow-up following DBS optimisation, broadly coinciding with improvements in sleep and quality-of-life measures. This case illustrates how wearable-derived data may complement conventional postoperative assessments by capturing longitudinal patterns not routinely measured in follow-up. Larger studies are required to determine whether such consumer-grade measures can be interpreted as clinically useful digital biomarkers.

## Introduction

Parkinson’s disease (PD) is a progressive neurodegenerative condition characterised by motor and non-motor symptoms, such as sleep disturbances [1]. Subthalamic nucleus deep brain stimulation (STN DBS) is an established therapy for motor fluctuations that can improve both motor and non-motor outcomes [2,3]. However, postoperative assessments are limited to intermittent clinic visits and may not reflect day-to-day function. This is clinically relevant as improvements in motor performance measured under controlled clinical conditions may not necessarily translate into better function in everyday life.

Traditional assessments, including the Movement Disorder Society-sponsored revision of the Unified Parkinson’s Disease Rating Scale (MDS-UPDRS) [4], and patient-reported measures, such as the 39-item Parkinson’s Disease Questionnaire (PDQ-39) [5,6], provide valuable but time-limited assessments and do not capture continuous fluctuations in activity, sleep, and functional response [7]. The MDS-UPDRS, for instance, primarily characterises clinical motor improvement and related symptoms, but does not measure how patients function between clinic visits. PD-specific wearable tools, including mPower, Parkinson’s KinetiGraph, and STAT-ON, have traditionally focused on monitoring motor symptoms and treatment response in PD [8-10], whereas consumer wearables offer the possibility of passively monitoring broader activity, sleep, and physiological patterns over prolonged periods. These non-motor and lifestyle-related outcomes remain less well characterised, particularly in DBS populations.

The Oura Ring is a multisensor smart ring demonstrating good usability for tracking sleep and physical activity in PD patients [11]. It combines technologies such as photoplethysmographic, inertial, and temperature sensors to generate three composite scores (0-100): the “Sleep Score”, reflecting sleep quality; the “Activity Score”, capturing daily activity patterns; and the “Readiness Score”, representing physiological preparedness, with higher scores reflecting more favourable health states. Validation studies of the individual raw signals have demonstrated that Oura-derived sleep metrics are comparable to polysomnography and activity metrics correlate with standard accelerometry [11-13]. Raw physiological metrics have shown high accuracy for nocturnal heart rate measurement relative to medical-grade electrocardiography [9]. However, the proprietary composite scores themselves have not been validated as clinical outcome measures and should therefore be interpreted as descriptive wearable-derived measures requiring validation before clinical application.

Despite increasing use of consumer wearables, their ability to provide complementary longitudinal information following DBS remains poorly understood. Here, we describe a patient who continuously wore an Oura Ring in the months surrounding STN DBS surgery for PD. The device was already owned and consistently used before surgery, allowing for uninterrupted data collection without additional study-related burden.

## Case presentation

Clinical course

A 68-year-old man with an 11-year history of PD presented with motor fluctuations, including prolonged OFF periods with freezing of gait, shuffling and hypophonia, alongside mild dyskinesia. A levodopa challenge demonstrated a 54% improvement in motor scores with medication (MDS-UPDRS Part III: 39 OFF medication, 18 ON medication). There were no cognitive, neuropsychiatric, or imaging contraindications to DBS. Baseline medication was 1,540 mg/day levodopa equivalent daily dose. The patient underwent bilateral STN DBS under general anaesthesia and recovered without complications. Continuous deep brain stimulation (cDBS) was initiated two days postoperatively and subsequently optimised during routine follow-ups.

Wearable data collection and analysis

The patient had consistently used an Oura Ring Generation 3 Horizon before surgery (since September 2022) and continued wearing the same device throughout follow-up. The patient’s wearable data were obtained with permission and analysed in two phases: a primary perioperative comparison and an extended longitudinal follow-up of up to 12 months. The month immediately before and after the surgery (day 0) was excluded to minimise confounding from perioperative restrictions. Primary analyses compared two predefined time windows: a preoperative “Pre-DBS” period (day -90 to day -30) and early postoperative “Post-DBS” period (day 30 to day 90). The 60-day windows were selected to provide sufficient observations while maintaining temporal proximity to surgery. Exploratory longitudinal analyses were performed across later postoperative timepoints alongside the clinical visits.

Oura Ring data were analysed in Python 3.13.1 (www.python.org). Duplicate dates were removed. Wearable data were available for 56/60 days (93.3%) during the preoperative period and 57/60 days (95.0%) during the early postoperative period. Missing values were not imputed for calculation of descriptive statistics; linear interpolation was used only for visualisation. Data were summarised descriptively, with composite scores reported using medians and interquartile ranges and daily step count summarised using the mean. Pre- and post-operative differences were visualised through a violin plot comparing “Sleep”, "Activity”, and "Readiness". The average daily step count was also compared across the observed periods. All comparisons were interpreted as descriptive within-subject contrasts intended to characterise pattern changes rather than generalisable effect estimates.

Clinical outcomes

At 2.5 months postoperatively, while on conventional cDBS, motor assessments showed marked improvement, with an approximately 75% improvement in OFF-medication MDS-UPDRS Part III (39 vs 10). However, fluctuations and quality of life measures worsened, with MDS-UPDRS Part IV increasing from 6 to 8 and the 39-item Parkinson’s Disease Questionnaire (PDQ-39) Summary Index increasing from 10.0 to 23.7.

At approximately 5.6 months postoperatively, during local field potential (LFP)-guided optimised cDBS, MDS-UPDRS Part IV, Parkinson’s Disease Sleep Scale-2 (PDSS-2) [14], and PDQ-39 Summary Index scores improved. At 7.6 months postoperatively, during adaptive DBS (aDBS), further improvements were observed. Levodopa equivalent daily dose (LEDD) decreased from 1540 mg/day preoperatively to 950 mg/day during aDBS. The Unified Dyskinesia Rating Scale (UDysRS) [15] was also recorded during clinical follow-up. Key clinical outcomes are summarised in Table 1.

**Table 1:** Clinical outcomes across key DBS follow-up timepoints aDBS: adaptive deep brain stimulation; ADL: activities of daily living; cDBS: continuous deep brain stimulation; LEDD: levodopa equivalent daily dose; LFP-cDBS: local field potential-guided continuous deep brain stimulation; MDS-UPDRS: Movement Disorders Society-sponsored revision of the Unified Parkinson’s Disease Rating Scale; NA: Not available/applicable; PDQ-39: 39-item Parkinson’s Disease Questionnaire; PDSS-2: Parkinson’s Disease Sleep Scale-2; UDysRS: Unified Dyskinesia Rating Scale.

Variable \ Visit	Pre-op	Visit 1	Visit 2	Visit 3
Time since surgery, months	NA	2.56	5.55	7.62
Stimulation mode	NA	Conventional cDBS	LFP-guided cDBS	aDBS
LEDD, mg/d	1540.00	1109.00	909.00	950.00
MDS-UPDRS part III, OFF medication	39.00	10.00	16.00	NA
MDS-UPDRS part IV	6.00	8.00	5.00	4.00
UDysRS	11.00	NA	0.00	1.00
PDSS-2 – Total score	13.00	NA	7.00	3.00
PDSS-2 – Motor symptoms at night	5.00	NA	0.00	1.00
PDSS-2 – PD symptoms at night	2.00	NA	1.00	0.00
PDSS-2 – Disturbed sleep	6.00	NA	6.00	2.00
PDQ-39 – Summary Index	10.00	23.66	8.70	3.01
PDQ-39 – Mobility	2.50	27.50	7.50	0.00
PDQ-39 – ADL	12.50	25.00	4.17	8.33
PDQ-39 – Emotional wellbeing	16.67	41.67	12.50	4.17
PDQ-39 – Stigma	6.25	12.50	25.00	6.25
PDQ-39 – Social support	0.00	8.33	8.33	0.00
PDQ-39 – Cognition	18.75	56.25	12.50	0.00
PDQ-39 – Communication	8.33	8.33	8.33	8.33
PDQ-39 – Bodily discomfort	25.00	33.33	0.00	0.00

Pre- vs. early post-operative period

The early post-DBS period was associated with an increase in “Sleep Score” from 45.0 pre-DBS to 53.5 post-DBS and in “Readiness Score” from 67.5 to 71.0. In contrast, overall activity remained largely unchanged, with minimal change in “Activity Score” and daily step count (Figure 1). Mean daily step count went from 11,662 steps pre-DBS to 11,283 steps post-DBS, suggesting that overall activity volume did not substantially change. This coincided clinically with ongoing stimulation adjustment and mixed questionnaire results, including worsening in MDS-UPDRS Part IV and PDQ-39 Summary Index despite improvement in MDS-UPDRS Part III.

**Figure 1:**
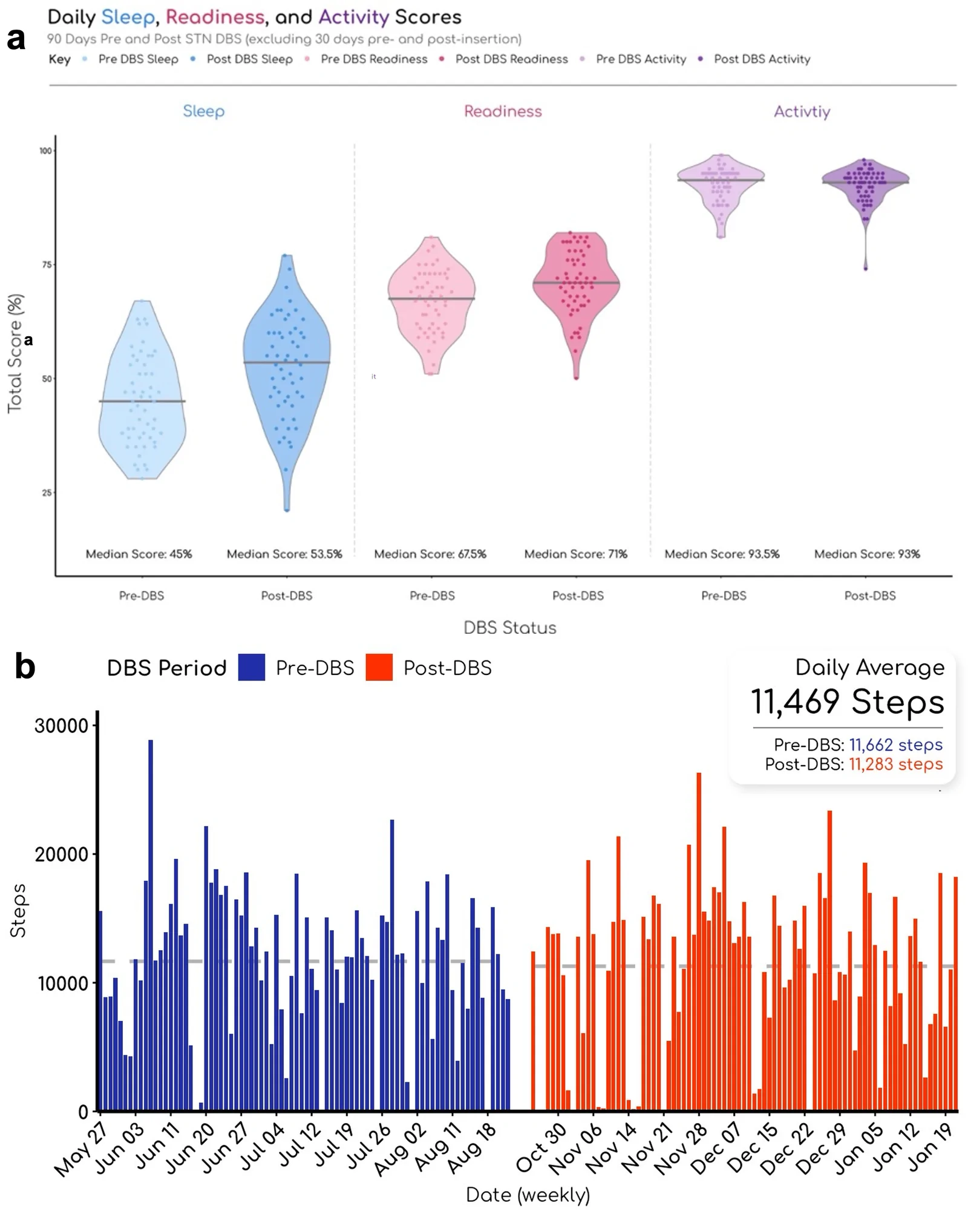
Perioperative Oura Ring metrics following bilateral STN DBS a) Violin plots showing the distribution of “Sleep”, “Readiness”, and “Activity” composite scores derived from the Oura Ring in the pre- and post-DBS periods. Scores were analysed over two 60-day windows before and after subthalamic nucleus deep brain stimulation (STN DBS), excluding the 30 days immediately before and after electrode implantation. Each dot in the figure represents the score on an individual day, and horizontal lines indicate median values. Composite scores range from 0-100, with higher values reflecting more favourable states. (b) Daily step counts during the pre- and post-DBS periods captured by the Oura Ring. Bars represent individual days within two 60-day windows before (blue) and after (orange) DBS implantation, excluding the 30 days immediately before and after surgery. Horizontal dashed lines indicate the mean daily step count for each period.

Extended postoperative period

Longitudinal analyses were used to examine trends in the Oura Ring metrics over 12 months postoperatively. Visual inspection of rolling averages suggested that “Activity Score” remained high overall, with intermittent fluctuations but no sustained change. In contrast, “Sleep” and “Readiness” scores increased during later follow-up, including during periods of cDBS and subsequent aDBS. Notably, “Sleep Score” showed a marked improvement during optimised cDBS (64.5 [IQR 56.3-70.8]) and aDBS (65.5 [IQR 54.5-71.0]) compared to both preoperative (45.0 [IQR 36.5-53.0]) and early postoperative periods (53.5 [IQR 46.8-60.0]). These longitudinal patterns were interpreted alongside clinical follow-up data. The early postoperative wearable window coincided with a period of stimulation adjustments and mixed questionnaire-based outcomes. In contrast, later periods during optimised cDBS and subsequent aDBS coincided with improvements in several clinical outcomes. The longitudinal wearable trends are shown in Figure 2.

**Figure 2:**
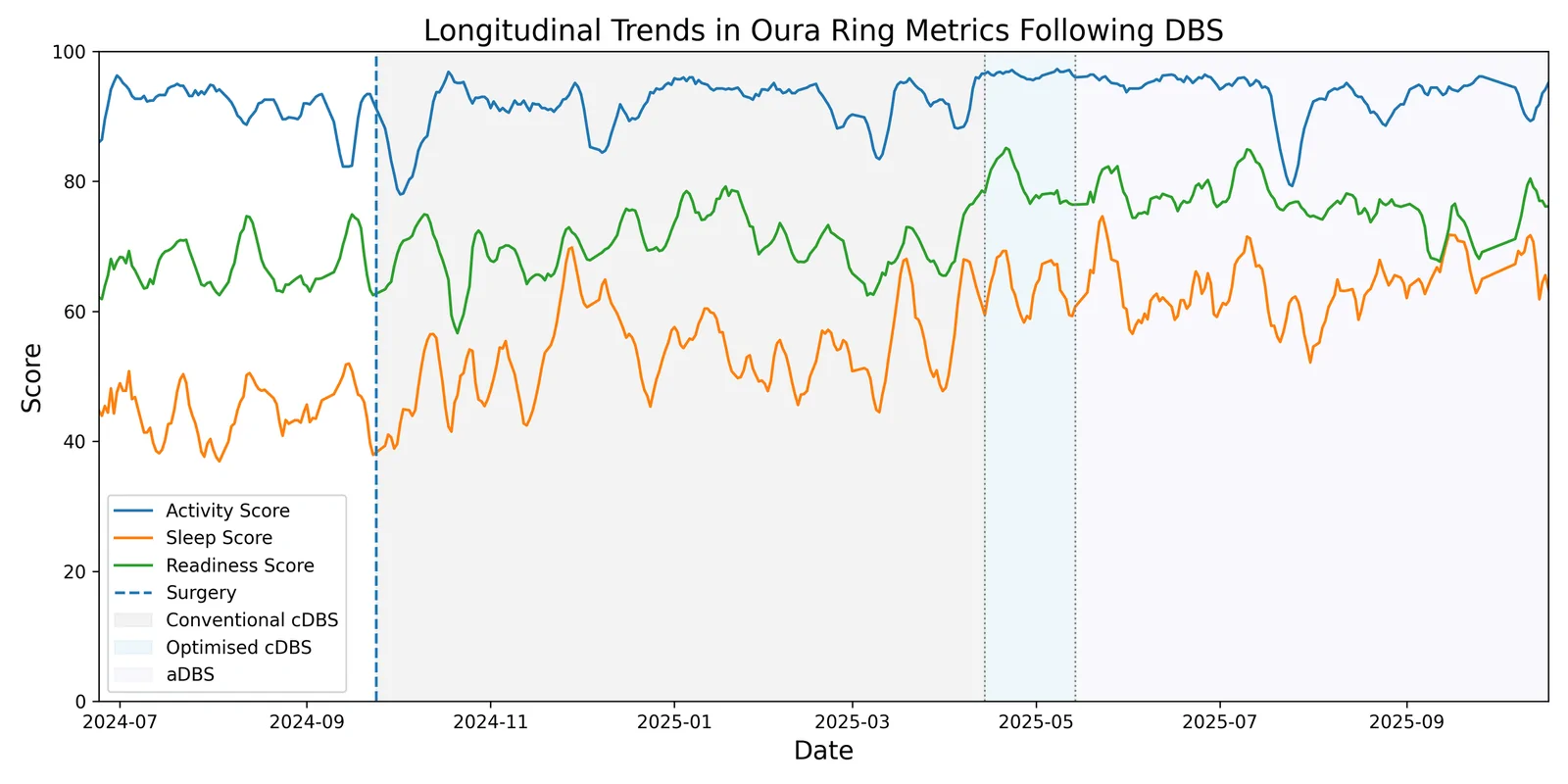
Longitudinal Oura Ring metrics following bilateral STN deep brain stimulation Longitudinal trends in Oura Ring metrics up to 12 months postoperatively are included. Seven-day rolling averages are shown for the Oura Ring composite scores (“Activity Score”, “Sleep Score”, and “Readiness Score”). The dashed vertical line indicates when the surgery was conducted. The shaded regions represent stimulation periods: conventional continuous deep brain stimulation (cDBS), optimised cDBS, and adaptive deep brain stimulation (aDBS). The proprietary composite scores range from 0 to 100, with higher values representing more favourable states. The stimulation periods are shown to provide temporal clinical context.

## Discussion

This case illustrates how continuous wearable-derived data may complement conventional clinical assessments by characterising activity, sleep and readiness following STN DBS. The predefined early post-DBS comparison found that Oura-derived “Sleep” and “Readiness” scores increased, while overall activity volume remained stable. This occurred despite improvement in the MDS-UPDRS Part III score, suggesting potential dissociation between clinic-based motor scores and day-to-day activity patterns.

This may reflect ceiling effects, habitual activity patterns, and ongoing adaptation to stimulation rather than lack of clinical benefit. The patient had high existing baseline step counts, and improved motor capacity does not necessarily translate into increased free-living activity, particularly in the absence of behavioural modification. The early postoperative period also coincided with stimulation adjustments and worsening quality of life (PDQ-39 Summary Index) and fluctuation (MDS-UPDRS Part IV) scores, despite motor improvement as reflected in MDS-UPDRS Part III scores.

These findings align with prior studies demonstrating that physical activity does not reliably increase following DBS, particularly in patients with axial symptoms [16]. Instead, activity patterns may be reorganised, with fewer but longer and more variable walking bouts and a concomitant increase in sedentary time [16-18]. Behavioural and motivational factors, including fatigue, fear of falling, habit formation and self-efficacy, may also influence free-living activity [16].

Longitudinal trends provided additional context alongside clinical follow-up. “Sleep Score” and “Readiness Score” further improved over time, broadly aligning with improvements in PDSS-2 and PDQ-39 scores. Improved Oura-derived sleep measures are consistent with previous studies reporting reductions in sleep-wake disturbances after STN DBS in PD [1,19]. Sleep represents a potential area of interest for aDBS, which may help prevent nocturnal overstimulation and maintain physiological sleep architecture [19]. However, further studies incorporating broader electrophysiological markers are needed to better characterise these effects [20]. In this case, it is also unclear whether observed changes in wearable-derived sleep measures reflected stimulation optimisation, medication change, behavioural adaptations, or other factors.

A key limitation of the present study is the single-patient design, which limits generalisability. Additionally, Oura composite scores are proprietary and have not been validated as clinical outcome measures in PD and DBS populations. The study also lacked directly comparable symptom-state data, limiting the ability to correlate data from the ring with motor states. Concurrent changes in stimulation settings, medication burden, and everyday behaviours may prevent attribution of longitudinal changes to a specific DBS mode. Findings should therefore be interpreted as descriptive and hypothesis-generating.

These limitations highlight the need for future studies incorporating larger cohorts, longer follow-up, validated clinical scales, and physiological measurements. Such studies could determine whether changes in consumer-grade wearable-derived metrics can reliably capture treatment response, behavioural change, or suboptimal stimulation settings beyond conventional follow-up.

## Conclusions

Overall, this case illustrates how continuous monitoring with a consumer wearable provided complementary longitudinal insights into “Activity”, “Sleep”, and “Readiness" following STN DBS. Improvement in clinic-based motor assessment was not accompanied by increased free-living activity in this patient, while “Readiness” and “Sleep” scores increased during longer-term follow-up, broadly coinciding with patient-reported outcomes. However, these temporal trends should not be interpreted as evidence that specific DBS modes caused the observed change. Larger prospective studies are required to validate wearable-derived measures in postoperative DBS monitoring against established clinical and physiological outcomes.

## References

[1] Sharma VD, Sengupta S, Chitnis S, Amara AW (2018). Deep brain stimulation and sleep-wake disturbances in Parkinson Disease: a review. Front Neurol.

[2] Koeglsperger T, Palleis C, Hell F, Mehrkens JH, Bötzel K (2019). Deep brain stimulation programming for movement disorders: current concepts and evidence-based strategies. Front Neurol.

[3] Tödt I, Al-Fatly B, Granert O (2022). The contribution of subthalamic nucleus deep brain stimulation to the improvement in motor functions and quality of life. Mov Disord.

[4] Goetz CG, Tilley BC, Shaftman SR (2008). Movement Disorder Society-sponsored revision of the Unified Parkinson's Disease Rating Scale (MDS-UPDRS): scale presentation and clinimetric testing results. Mov Disord.

[5] Peto V, Jenkinson C, Fitzpatrick R, Greenhall R (1995). The development and validation of a short measure of functioning and well being for individuals with Parkinson's disease. Qual Life Res.

[6] Jenkinson C, Fitzpatrick R, Peto V, Greenhall R, Hyman N (1997). The Parkinson's Disease Questionnaire (PDQ-39): development and validation of a Parkinson's disease summary index score. Age Ageing.

[7] Lim SY, Tan AH (2018). Historical perspective: the pros and cons of conventional outcome measures in Parkinson's disease. Parkinsonism Relat Disord.

[8] Rodríguez-Martín D, Cabestany J, Pérez-López C (2022). A new paradigm in Parkinson's Disease evaluation with wearable medical devices: a review of STAT-ON(TM). Front Neurol.

[9] Cao R, Azimi I, Sarhaddi F, Niela-Vilen H, Axelin A, Liljeberg P, Rahmani AM (2022). Accuracy assessment of Oura ring nocturnal heart rate and heart rate variability in comparison with electrocardiography in time and frequency domains: comprehensive analysis. J Med Internet Res.

[10] Powers R, Etezadi-Amoli M, Arnold EM (2021). Smartwatch inertial sensors continuously monitor real-world motor fluctuations in Parkinson's disease. Sci Transl Med.

[11] Reithe H, Marty B, Torrado JC (2025). Cross-evaluation of wearable data for use in Parkinson's disease research: a free-living observational study on Empatica E4, Fitbit Sense, and Oura. Biomed Eng Online.

[12] Kristiansson E, Fridolfsson J, Arvidsson D, Holmäng A, Börjesson M, Andersson-Hall U (2023). Validation of Oura ring energy expenditure and steps in laboratory and free-living. BMC Med Res Methodol.

[13] Khan S, Ibrahim AF, Vasudevan SS, Quatela OE, Nanu DP, Carr MM (2025). The Oura ring versus medical-grade sleep studies: a systematic review and meta-analysis. OTO Open.

[14] Trenkwalder C, Kohnen R, Högl B (2011). Parkinson's disease sleep scale-validation of the revised version PDSS-2. Mov Disord.

[15] Goetz CG, Nutt JG, Stebbins GT (2008). The Unified Dyskinesia Rating Scale: presentation and clinimetric profile. Mov Disord.

[16] Danoudis M, Iansek R (2023). Physical activity levels in people with Parkinson's disease treated by subthalamic nucleus deep brain stimulation. Disabil Rehabil.

[17] Rochester L, Chastin SF, Lord S, Baker K, Burn DJ (2012). Understanding the impact of deep brain stimulation on ambulatory activity in advanced Parkinson's disease. J Neurol.

[18] Jorgensen HU, Werdelin L, Lokkegaard A, Westerterp KR, Simonsen L (2012). Free-living energy expenditure reduced after deep brain stimulation surgery for Parkinson's disease. Clin Physiol Funct Imaging.

[19] Amara AW, Walker HC, Joop A, Cutter G, DeWolfe JL, Harding SM, Standaert DG (2017). Effects of subthalamic nucleus deep brain stimulation on objective sleep outcomes in Parkinson's disease. Mov Disord Clin Pract.

[20] Carver K, Saltoun K, Christensen E, Abosch A, Zylberberg J, Thompson JA (2023). Towards automated sleep-stage classification for adaptive deep brain stimulation targeting sleep in patients with Parkinson’s disease. Commun Eng.

